# Extracellular vesicles as mediators of prostatic diseases: connecting function to diagnostic and therapeutic applications

**DOI:** 10.20517/evcna.2025.96

**Published:** 2026-04-22

**Authors:** Julie Bishara, Morolake Morakinyo, Sharanjot Saini

**Affiliations:** Department of Biochemistry and Molecular Biology, Augusta University, Augusta, GA 30912, USA.

**Keywords:** EVs, exosomes, prostate cancer, BPH, therapy, biomarkers

## Abstract

Diseases of the prostate gland, including benign prostatic hyperplasia (BPH) and prostate cancer (PCa), affect a significant proportion of men worldwide. The incidence of these diseases increases with advancing age decreased quality of life and mortality in cases of aggressive PCa. Advanced PCa shows a spectrum of disease states, including castration-resistant prostate cancer (CRPC) and therapy-resistant neuroendocrine prostate cancer (NEPC). NEPC is a highly aggressive, AR-independent state that evolves from CRPC by “lineage switching”. Although the underlying cellular mechanisms have been examined extensively, the role of extracellular-mediated intercellular communication is less well understood. Emerging evidence suggests an important role of extracellular vesicles (EVs) in the pathobiology of these diseases. EVs have been shown to be critical for imparting tumor aggressiveness via inducing epithelial to mesenchymal transition (EMT), stemness and immune evasion. Understanding EV-mediated signaling that governs the progression of prostatic diseases is crucial for developing effective targeted therapies and robust biomarkers for prognosis and risk stratification. Advances in EV engineering have enabled the development of targeted exosome-based therapeutics capable of delivering drugs, biologic payloads, or immune-stimulating signals. In this article, we review our current knowledge on the role of EVs in prostatic diseases, explore their diagnostic and therapeutic applications, and outline future directions aimed at translating EV-based technologies into tools for improved clinical management.

## INTRODUCTION

**Figure 1:**
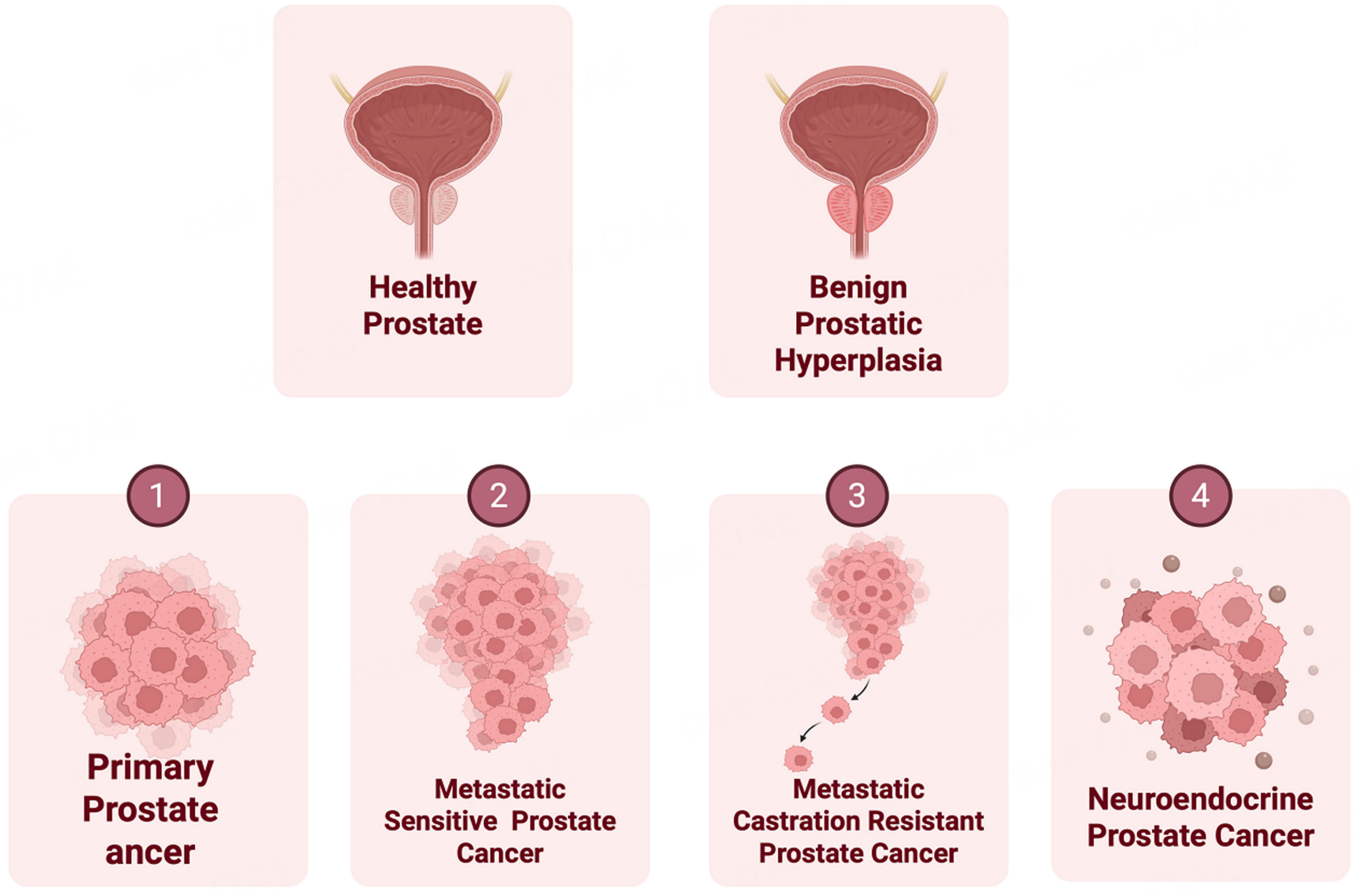
Schematic representation showing normal prostate, BPH and spectrum of PCa disease states. Upper panels: Normal healthy prostate and prostate gland with BPH. Lower panels: Progressive stages of prostate cancer are depicted. Created in BioRender. Bishara, J. (2026) https://BioRender.com/zwoiqpc. BPH: Benign prostatic hyperplasia; PCa: prostate cancer.

Prostatic diseases affect a significant proportion of men worldwide, impairing quality of life, particularly in aging populations. These diseases encompass a wide range of conditions, from benign prostatic hyperplasia (BPH) to aggressive forms of prostate cancer (PCa) [Figure 1]. BPH is a non-malignant enlargement of the prostate gland, often associated with lower urinary tract symptoms (LUTS) caused by obstruction of the bladder outlet^[1-3]^. While BPH is not cancerous and is not a precursor for PCa, androgen signaling, inflammation, and reactive stroma^[4,5]^ are common determinants of BPH and malignant transformation, underscoring the complex biology of the prostate.

PCa is the second most common male cancer and is a leading cause of cancer-related mortality among men. An estimated 313,780 new cases are projected for 2025 in the United States^[6]^, and over 10% of these cases are expected to result in mortality^[7]^. Androgens acting via Androgen Receptor (AR) signaling fuel oncogenic transformation in PCa. Therefore, ablation of AR signaling by androgen deprivation therapy (ADT) is often used as a first-line therapy^[8]^ that initially results in cancer regression. However, prolonged hormonal therapy inevitably leads to castration-resistant prostate cancer (CRPC)^[9]^ [Figure 1] that has limited therapeutic options. The treatment landscape of CRPC has significantly evolved in recent years with the introduction of a newer generation of AR pathway inhibitors (APIs) such as Enzalutamide (ENZ) and abiraterone (ABI) as a second line of therapy. These agents are used for men with metastatic and non-metastatic CRPC, which leads to improved survival. However, a significant fraction of CRPC patients develop drug resistance over time, resulting from heterogeneous molecular alterations leading to the emergence of alternate disease states. At the molecular level, alterations such as AR amplification, emergence of ligand-independent splice variants, AR bypass signaling, or complete AR independence^[3,4]^ underlie these states. One of the AR-independent pathways include the emergence of neuroendocrine prostate cancer (NEPC), wherein PCa cells undergo lineage switching referred to as “neuroendocrine differentiation (NED)” [Figure 1]. As a result of NED, PCa cells exhibit decreased expression of classical luminal lineage markers such as AR and prostate-specific antigen (PSA) coupled with increased expression of neuroendocrine (NE) lineage markers such as enolase 2 (ENO2), chromogranin A (CHGA) and synaptophysin (SYP)^[10,11]^. NEPC is a highly aggressive PCa variant with survival rates < 1 year. The primary clinical characteristics of NEPC are its aggressive phenotype with rapid disease progression, soft tissue metastases, and a poor prognosis despite low serum PSA. Current treatment options for NEPC include platinum-based chemotherapy^[12]^. However, high toxicity levels have been a recognized drawback of this therapeutic approach^[13]^. Despite some responsiveness, there is no established clinical standard for NEPC care, and treatment outcomes remain suboptimal^[12]^. Furthermore, there is a lack of effective molecular biomarkers for predicting API therapy resistance and the emergence of therapy-induced NEPC^[10,11]^ in CRPC patients. Though histopathological analyses and neuronal markers, including SYP, NSE, CHGA, and cluster of differentiation 56 (CD56), have been used to monitor API-induced NEPC in biopsy tissues or serum samples^[11]^, these methods are not highly specific^[14]^. In addition, obtaining biopsies from CRPC patients is not feasible due to the invasiveness of the procedure, highlighting the urgent need for non-invasive molecular biomarkers for NEPC.

Understanding the cellular and molecular mechanisms that govern the progression of prostatic diseases, including BPH, primary PCa, metastatic advanced PCa, and the emergence of therapy-induced NEPC, is crucial for developing effective targeted therapies and robust biomarkers for prognosis and risk stratification. Emerging evidence suggests an important role of extracellular vesicles (EVs), such as exosomes, in the pathobiology of these prostatic diseases. Recent studies in BPH have shown that EVs secreted by prostatic epithelial and stromal cells can influence tissue remodeling, inflammation, and cellular proliferation, key features of BPH pathobiology. In the context of PCa, particularly CRPC and NEPC, it has been recognized that EVs serve as critical mediators of tumor progression, immune evasion, and therapy resistance. Furthermore, given the fact that EV cargo often reflects the molecular signature of originating cells, EVs have tremendous potential as non-invasive biomarkers for disease diagnosis and monitoring of disease progression and treatment outcomes. Several promising EV-based RNA/miRNA/protein biomarkers have been identified for prostate PCa that predict disease progression, drug resistance, and immune responses. Furthermore, EVs has been exploited for the treatment of various prostatic diseases ranging from BPH to aggressive PCa including CRPC and NEPC. For therapeutic applications, EVs have been used to deliver cytotoxic payloads such as chemotherapeutic drugs or biologics. In addition, an important immunomodulatory role of EVs have been recently recognized and EVs are being evaluated for modulating immune responses in PCa. This review article explores the evolving role of EVs in BPH and PCa, with a special focus on their contribution to CRPC/NEPC progression and their promise as liquid biopsy biomarkers and therapeutic delivery vehicles. We will highlight major findings in this area, over last few years that have potential translational implications.

### Heterogeneity of EVs: many particles with diverse roles

**Figure 2:**
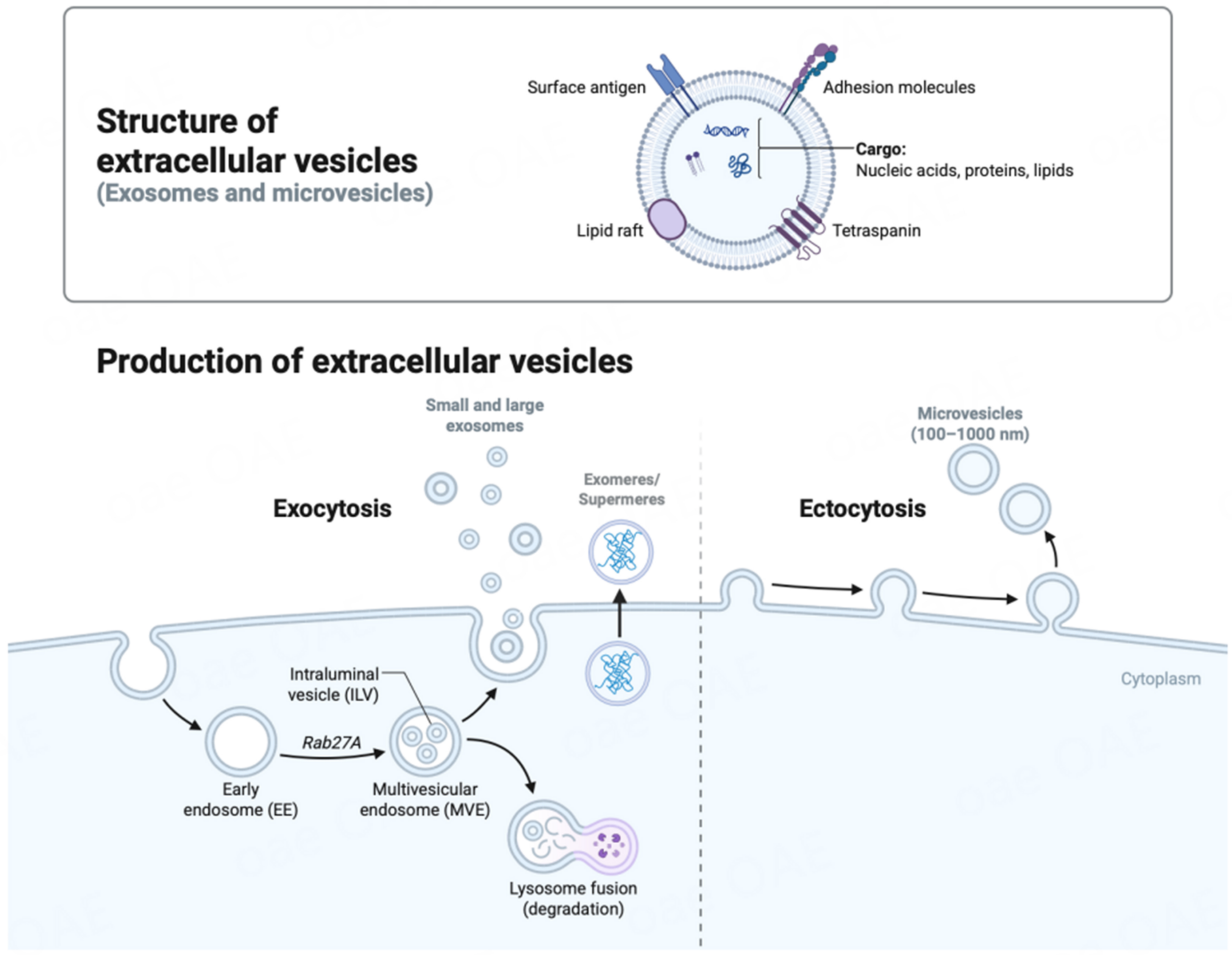
A schematic representation showing biogenesis of major subtypes of EVs. Microvesicles are generated through outward budding of the plasma membrane via ectocytosis while exocytosis releases small and large exosomes. Exosomes originate from the endosomal pathway via MVBs/late endosomes. MVBs are either sorted for lysosomal degradation or leads to the formation of ILVs that are transported to the plasma membrane and released as small/large exosomes. The structure of a typical exosome is schematically represented. In addition, exomeres or supermeres are released as non-membranous particles (< 50 nm) containing nucleic acids, lipids and proteins. Created in BioRender. Saini, S. (2026) https://BioRender.com/vr7efu4. EVs: Extracellular vesicles; ILV: intraluminal vesicle; MVB: multivesicular body; MVE: multivesicular endosome; EE: early endosome; Rab27A: Ras-related protein Rab-27A.

EVs are nano-sized vesicles secreted by all cell types that serve as a mode of intercellular communication. These vesicles carry various cargo, such as lipids, proteins, RNA/miRNA, DNA and metabolites that are specifically packaged and released by originating cells [Figure 2]. EVs are a highly heterogenous population of vesicles that include a wide variety of particles, ranging from 10 nm-1 µm in diameter. Since their discovery in the 1980s, research over the last few years has shown that cells release an array of vesicles- ranging from supermeres (25-35 nm), exomeres (35-50 nm), exosomes (typically between 30-150 nm in size), microvesicles. (50-2,000 nm), apoptotic bodies (800-5,000 nm), and a host of other vesicles^[15]^. Conventionally, EVs can be differentiated into exosomes and microvesicles^[16]^ based on their biogenesis, cargo, release pathways, size, and primary purpose^[16]^ [Figure 2]. Exosomes, the most widely studied vesicles, are nano-sized (30-150 nm in size) membranous EVs^[17]^ that are released by all living cells. Exosomes are formed inside multivesicular bodies (MVBs) in the cells’ endosomal system, which undergo further maturation within the cytoplasmic environment^[18]^. Based on their size, exosomes have been categorized into large (Exo-L, 90-150nm) and small exosomes (Exo-S, 60-90 nm). Smaller particles such as exomeres and supermeres differ from exosomes as they lack a lipid bilayer and therefore classified as extracellular nanoparticles (ENPs) [Figure 2]. Exosomes^[17]^ carry a cargo of RNA, proteins and lipids and facilitate intercellular communication by transferring their cargo to recipient cells to modulate target cell functions^[19,20]^. The study of exosomes has gained remarkable traction in cancer biology, specifically for their roles in tumor progression, metastasis, and intercellular communication^[15]^. Exosomes are released by most cell types, including cancer cells, and act as vital intermediaries of cell-to-cell signaling. These vesicles contain a cargo of bioavailable molecules - such as proteins, lipids, and nucleic acids - that reveal the physiological and pathological state of the parent cell, making them a significant source of cancer biomarkers and therapeutic targets^[21]^.

Given their potential to carry a repertoire of molecules, exosomes have been effectively exploited for therapy of various diseases. Owing to their biocompatibility, low toxicity, low immunogenicity, high permeability, and stability in biological fluids, exosome-based therapies have several advantages. Furthermore, exosomes can be effectively engineered to carry drugs to specific sites for targeted drug delivery. Apart from exosomes, oncosomes - a specialized subclass of EVs - have attracted a lot of attention in the field of PCa. While oncosomes and exosomes are both EVs involved in intercellular communication, the two types of EVs differ in a variety of ways: primarily, their differences in size, origin, and function highlight their distinct roles in physiological and pathological processes^[22]^ [Table 1]. Oncosomes are tumor-derived and are characterized by their large size relative to exosomes, typically ranging from 1 to 10 µm in diameter^[23]^. These EVs are released almost exclusively by cancer cells, particularly those undergoing a transformation into an ameboid phenotype^[24]^. This phenotype is induced by factors including an overexpression of oncogenes or the silencing of cytoskeletal regulators such as DIAPH^[21]^. Like exosomes, oncosomes carry a diverse set of cargo, including nucleic acids, lipids, and proteins, thus revealing the molecular properties of their parent tumor cells. Remarkably, oncosomes have been found to accommodate bioactive molecules like metalloproteases, specific microRNAs, and caveolin-1, which can regulate the tumor microenvironment^[24]^ and therefore PCa progression^[23]^.

**Table 1:** Comparison of oncosomes and exosomes as subclasses of extracellular vesicles

**Feature**	**Oncosomes**	**Exosomes**
Size	1-10 μm	30-150 nm
Origin	Plasma membrane blebbing^[23]^	Endosomal pathway (multivesicular bodies)^[17]^
Cellular source	Predominantly cancer cells^[23]^	Most cell types
Cargo	Proteins, DNA, RNA, lipids	Proteins, RNA, lipids
Function	Modulate tumor microenvironment, promote metastasis	Intercellular communication, antigen presentation
Detection	Visible via light microscopy	Requires ultracentrifugation and electron microscopy

The complexity of EV subpopulations and classification are constantly evolving. The International Society of Extracellular Vesicles (ISEV) has provided a set of guidelines referred to as “Minimal Information for Studies of EVs (MISEV)” to provide a standardized set of reporting criteria for EVs^[25]^. These guidelines are periodically updated to encompass the increasing knowledge of these highly heterogeneous vesicles. While the biogenesis, functions, and target cells for these vesicles are being examined, it has been widely recognized that EVs play an indispensable role in various physiological and pathological processes, such as cancer^[15]^.

### EVs in PCa: important roles in progression, metastasis and immune responses

**Figure 3:**
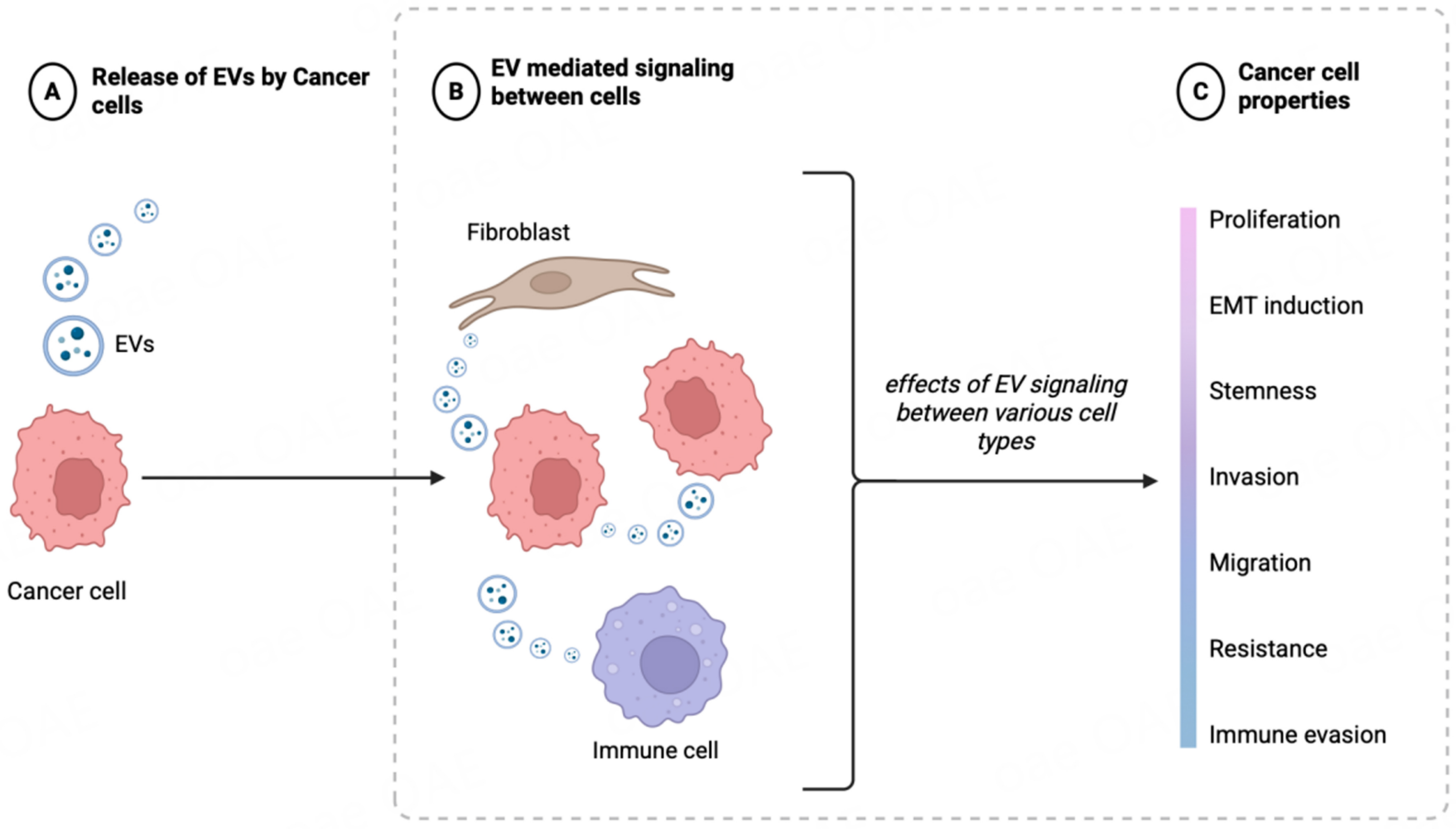
Schematic representation showing the important role of EVs in prostate cancer: progression, metastasis and immune responses. EV-mediated signaling between PCa cells or with cells of TME contributes to PCa cell attributes such as proliferation, EMT/stemness, invasion, migration, drug resistance or immune evasion. Created in BioRender. Saini, S. (2026) https://BioRender.com/vr7efu4. EVs: Extracellular vesicles; PCa: prostate cancer; TME: tumor microenvironment; EMT: epithelial-mesenchymal transition.

Studies have shown that EVs contribute to multiple stages of PCa progression by modulating the tumor microenvironment (TME)^[26,27]^, influencing immune responses, promoting angiogenesis, and promoting the formation of pre-metastatic niches (PMN), thereby aiding in tumor growth and dissemination^[15]^ [Figure 3]. A recurring theme that has emerged from mechanistic studies is that EV cargo from more aggressive PCa cells can reprogram less aggressive PCa cells towards a more invasive/epithelial to mesenchymal transition (EMT) and stem-like state^[27]^. Tumor secreted vesicles can transfer signaling molecules between tumor cells within the primary tumor and also contribute to invasion and organotropism of metastatic tumors^[28]^. Caveolin-1 contained in PCa cell-derived EVs was shown as a potent driver of cancer stem cell (CSC) phenotypes and EMT through NFκB signaling pathway. Caveolin-1 containing exosomes were also found to induce chemo-resistance and radio-resistance in recipient cells^[29]^.

EVs have been shown to carry important RNAs/proteins that have been implicated in PCa progression and metastasis. Androgen Receptor Variant 7 (AR-V7), a splice variant of AR that lacks the C-terminal ligand binding domain, is associated with PCa progression and drug resistance. AR-V7 was found to be present in tumor tissues, circulating tumor cells (CTCs), or in blood mRNA^[30-32]^. Its levels are associated with resistance to APIs such as ENZ and ABI. EVs released by mesenchymal-like PCa cells were shown to modulate the EMT state of recipient epithelial-like PCa cells through reducing AR signaling and activating the TGFβ pathway^[33]^. Tumor suppressor PTEN has been shown to be released in PCa EVs, and its release in EVs mirrored the PTEN status of originating cells, suggesting that EVs not only carry tumor suppressor genes but also participate in the intercellular modulation of PTEN function within the tumor microenvironment (TME)^[34]^.

EVs have been implicated in bone-tropism and metastasis wherein functional studies suggest that EVs educate bone stromal cells [Figure 3]^[35]^. PCa exosomes promote bone metastasis by influencing the differentiation of osteoclasts^[36]^ and osteoblasts^[37]^. Dai *et al.* elegantly demonstrated the role of prostate tumor derived EVs in educating bone stroma through transfer of pyruvate kinase M2 (PKM2) in EVs^[35]^. PKM2 up-regulated CXCL12 production in a HIF-1α-dependent fashion in bone marrow stromal cells, which enhanced PCa seeding and growth in the bone marrow. PCa cell-secreted miR-940 in EVs, in turn promoted osteoblastic phenotype in the bone metastatic microenvironment^[37]^.

Importantly, EVs have been implicated in PCa immune evasion [Figure 3]. Recently, it was reported that PCa secretes PD-1 in exosomes that enhanced the activity of myeloid-derived suppressor cells (MDSCs) by activating JAK-STAT3 signaling^[38]^. Activated MDSCs in turn promoted immune evasion by reducing the infiltration of CD8+ T-cells^[38]^. B7-H3 (CD276), a member of the B7 family of immune-regulatory ligands, is an attractive therapeutic target for PCa. A recent study demonstrated that B7-H3 is associated with EVs, with its expression increasing in CRPC as compared to castration-sensitive PCa (CSPC)^[39]^. Overall, these studies highlight an important role of EVs in PCa progression and metastasis via influencing on various steps in the metastatic cascade.

### An emerging role of EVs in NEPC

**Figure 4:**
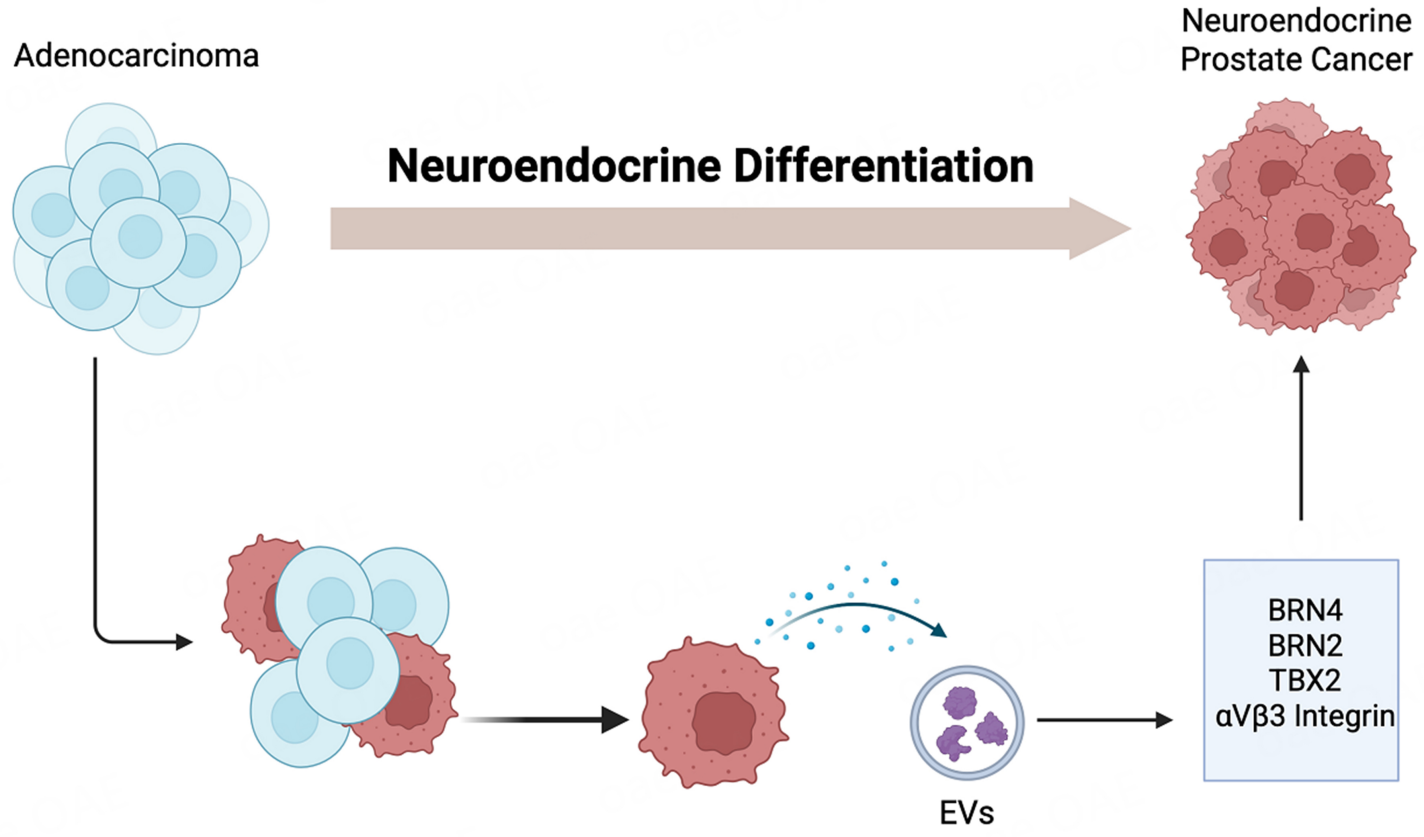
Schematic representation showing the role of EVs in emergence of neuroendocrine prostate cancer. Studies have shown that EVs carry functional factors such as BRN4, BRN2, TBX2 and specific integrins that when up taken by recipient cell modulate gene programs in recipient cells promoting NEPC. Created in BioRender. Saini, S. (2026) https://BioRender.com/e5v4yy5. EVs: Extracellular vesicles; NEPC: neuroendocrine prostate cancer; BRN2: brain-specific homeobox/POU domain protein 2; BRN4: brain-specific homeobox/POU domain protein 4; TBX2: T-box transcription factor 2.

It is now being recognized that the process of “lineage switching” leading to the emergence of NEPC following AR-targeted therapies is a highly complex process, driven by a multitude of genetic and epigenetic alterations^[40-44]^. The key genetic events driving this transition include the dual loss of the tumor suppressors retinoblastoma (*RB1*) and tumor protein 53 (*TP53*) coupled with alterations in phosphatase and tensin homolog (*PTEN*)^[40-44]^. Emerging evidence suggests that NEPC arises via clonal evolution from adenocarcinoma states, driven by altered transcriptomic landscapes resulting from dysregulation of key transcription factors (TFs). Key TFs, including Achaete-scute family bHLH transcription factor 1 (ASCL1), Neurogenic differentiation 1 (NEUROD1), Forkhead box protein A2 (FOXA2), and Brain-2 (BRN2), have been implicated in NEPC. Complementing the studies showing molecular heterogeneity of NEPC states, recent studies highlight an important role of EV-mediated communication in driving lineage plasticity in PCa [Figure 4]. NE-like cells in prostatic tumors have been known to possess the potential to enhance androgen-independent tumor growth in a paracrine manner by providing growth and survival signals to surrounding tumor cells, thereby contributing to the progression of the disease. Deeble *et al.* demonstrated that LNCaP cells induced to express a constitutively activated form of the cyclic AMP-dependent protein kinase A catalytic subunit (caPKA) exhibited NE features with loss of mitotic activity, and that these cells had the potential to enhance the growth of co-cultured prostate tumor cells in a paracrine manner under androgen deprivation^[45]^. An increasing body of recent evidence implicates an important role of EV-mediated communication in driving PCa NED [Figure 4]. Lin *et al*. demonstrated that adipocyte differentiation-related protein (ADRP) was released in exosomes during IL-6-induced NED in DU145 and C4-2 PCa cell lines that were capable of inducing NED in a paracrine manner^[46]^. In another study, TBX2 was reported to drive PCa NED via intercellular EV-mediated communication. They found that the effects of TBX2 on NED are mediated via miR-200c-3p, a tumor suppressor miRNA, and its release in EVs. These EVs are taken up by recipient cells, leading to upregulation of miR-200c-3p targets, sex determining region Y-box 2 (SOX2), and MYCN, facilitating an aggressive NEPC phenotype^[47]^. Another group has established an important role of small EVs (sEVs) in driving NED via release of integrin αVβ3 that mediates PCa NED via binding to a receptor NgR2 in recipient cells^[48]^ [Figure 4]. Large EVs (LEVs) were recently shown to reprogram anti-tumor immunity via stimulation of IFN signaling and expansion of specialized CD8+ T-cells owing to release of HMGB1 in these vesicles^[49]^. Packaging of HMGB1 in LEVs was, in turn, controlled by Parkin, a mitochondria-associated E3 ubiquitin ligase involved in organelle quality control via mitophagy^[50]^.

### Authors’ contributions to the field

We hypothesized that EVs mediate intercellular signaling in NEPC and play a role in oncogenic reprogramming of CRPC-Adeno to CRPC-NE states via the transfer of functional factors. Our studies identified BRN4, a POU-domain transcription factor, as a novel driver of NED in CRPC^[51]^.

Our findings suggest that BRN4 is specifically overexpressed in NEPC samples. Mechanistically, BRN4 collaborates with BRN2, another transcription factor previously implicated in NEPC, to promote NED by regulating SOX2^[52]^. Notably, we found that both BRN4 and BRN2 (mRNA and proteins) are actively released in PCa EVs, upon treatment with APIs like ENZ. Importantly, treatment of ENZ-resistant LNCaP-AR cell line with EV inhibitor GW4869 could partially restore the sensitivity of these cells to ENZ, suggesting a potential contribution of EVs in ENZ responsiveness These findings are in line with other studies showing a role of EV secretion contributing to the survival of ENZ resistant PCa cells^[53]^. These findings lend support to the hypothesis that EVs are crucial to NED induction and that key oncogenic factors, including *BRN2* and *BRN4,* are released in PCa EVs [Figure 4] and that that EV-associated BRN4 and BRN2 are horizontally transferred to neighboring cancer cells to propagate NED states.

These studies highlight an important emerging role of different populations of EVs in driving the complex process of PCa NED with potential translational implications.

### EV-based biomarkers for PCa

A typical PCa biomarker is serum PSA, a protein expressed by prostate epithelial cells. PSA levels typically increase in PCa, leading to its secretion into the blood, qualifying serum PSA as a PCa biomarker. Serum PSA has been employed as a PCa biomarker for more than three decades, for disease diagnosis and treatment monitoring^[8]^. However, PSA has inherent limitations, including a lack of specificity to PCa, as its levels can be elevated in BPH, prostatitis, or infections. Owing to these limitations, PSA based screening has led to overdiagnosis and over-treatment of PCa, leading to longstanding controversy regarding PSA-based screening. Consequently, there has been substantial interest in alternative PCa biomarkers, particularly those that can predict disease aggressiveness and aid in patient stratification for clinical decision making. Over the past few years, EVs have gained significant attention as a source of alternative PCa biomarkers^[54]^. EVs carry diverse biomolecular cargo- including RNA, DNA, proteins, lipids and metabolites. Due to a diverse set of biomolecules carried in EVs, these vesicles hold promise as “liquid biopsy-based” multi-faceted biomarkers for PCa diagnosis, therapeutic monitoring, and the emergence of resistant states. Several studies have investigated and compared EV profiles in serum/plasma/urine of PCa patients and control subjects^[55]^. These studies have identified several EV-based biomarkers in PCa.

Importantly, PSA itself has been shown to circulate in association with EVs, with EV bound PSA comprising a greater proportion of total PSA, particularly with serum PSA levels < 4 μg/L^[56-58]^. A recent study systematically analyzed the impact of the association of PSA with EVs on total PSA measurement employing several commercial PSA assays^[59]^. This study suggests that total PSA measurements obtained with different kits are differentially influenced by EV-associated PSA, warranting a more careful consideration of EV bound PSA in relation to total PSA for clinical use^[59]^.

Over the past decade, and particularly within the last five years, multiple prospective clinical trials and validation studies have established EVs as a clinically relevant biomarker platform in PCa. The clinical relevance of EV-based biomarkers is exemplified by ExoDx Prostate (Intelliscore), the first, EV-based, non-invasive diagnostic test that is Food Drug and Administration (FDA) approved, a Clinical Laboratory Improvement Amendments (CLIA)-certified, non-invasive EV-based diagnostic test integrated into PCa risk stratification algorithms. This urine-based test quantifies the expression level of 3 genes - *ERG*, *PCA3* and *SPDEF* - in urinary EVs to generate a risk score (1-100) predicting the likelihood of clinically significant PCa (Gleason score ≥ 7) in men aged ≥ 50 years with PSA 2-10 ng/mL, without requiring a digital rectal exam (DRE)^[60]^. This assay has been shown to not only provide individualized PCa risk assessment at initial biopsy but was found to be relevant on in subsequent repeat biopsies^[61]^. An ongoing clinical trial (NCT06966089) is studying the use of ExoDx Intelliscore in Magnetic Resonance Imaging (MRI)-negative men with high PSA. Beyond early detection, emerging clinical trials of urine and blood-based EV assays increasingly focus on prediction of aggressive PCa leveraging EV RNA, miRNA, protein, and surface-marker signatures to improve discrimination of high-grade tumors compared with PSA alone^[62]^. These clinical trials include Clarity Dx (NCT06678828) and Sentinel^TM^ PCC4 Assay (NCT04100811). The Sentinel PCC4 assay utilizes a set of 442 small non-coding RNAs extracted from urinary EVs to provide an indication of disease status in men with suspicion of PCa. ClarityDx Prostate is based on the utility of total and free PSA as well as simple clinical features to determine a patient’s risk of having clinically significant PCa.

Beyond diagnostic applications, EVs have been shown to carry biomarkers associated with therapy resistance and PCa progression. AR-V7, a splice variant of AR, which lacks the C-terminal ligand binding domain, and confers resistance to APIs, has been detected in tumor tissues, CTCs and in blood-derived RNA^[30-32]^. Del Re *et al*. showed that AR-V7 is associated with plasma EVs and can be a predictive biomarker of resistance to hormonal therapy^[63]^. AR-V7 has also been detected in urinary EVs from CRPC patients at higher levels as compared with those from hormone-sensitive disease^[64]^, although comparative analyses suggest higher detection frequencies in CTCs relative to EVs^[65]^. EV biomarkers are being incorporated as exploratory and secondary endpoints in therapeutic clinical trials, where circulating EV-associated markers such as AR-V7 transcripts and Prostate Specific Membrane Antigen (PSMA)-positive EVs are evaluated for their ability to predict treatment resistance, tumor burden, and clinical outcomes (e.g., NCT03694483).

Other EV-associated mRNA and protein biomarkers have likewise been extensively investigated^[62,66]^. B7-H3 (CD276), an immune checkpoint molecule and emerging therapeutic target in PCa, has been identified in EVs derived from PCa cells, with increased EV-associated B7-H3 observed in CRPC as compared to castration-sensitive disease. Elevated levels of B7-H3 correlate with poor clinical outcomes, supporting its prognostic utility in liquid biopsy-based assays^[39]^. More recently, Li *et al*. conducted a prospective multicenter study of urinary PCa EVs wherein a combination of PSA, PSMA, and Alpha-methylacyl-CoA racemase (AMACR) was shown to have an excellent diagnostic accuracy for PCa detection^[67]^.

Proteomic studies over the last decade have established urinary EVs as a valuable, non-invasive source of biomarkers for PCa detection, prognosis, and disease aggressiveness^[62,66]^. Park *et al.* isolated PCa EVs from urine and plasma using a combination of surface markers PSA and PSMA^[68]^. These prostate-specific EVs were quantifiable and could distinguish between healthy controls, BPH, and PCa patients, with abundance and molecular content correlating with tumor stage and Gleason score^[68]^. Overbye *et al*. performed large-scale urinary exosome proteome profiling using liquid chromatography–tandem mass spectrometry (LC-MS/MS) and identified that combined expression of transmembrane protein 256 (TMEM256) and late endosomal/lysosomal adaptor, MAP Kinase, and mechanistic target of rapamycin (mTOR) activator (LAMTOR1) could detect PCa with high sensitivity (AUC = 0.94)^[69]^. Sequeiros T examined a panel of protein biomarkers to distinguish between benign and PCa patients and identified a combination of two proteins, Adseverin (ADSV) and Transglutaminase4 (TGM4), to have good discriminatory power. They further discovered that a combination of five proteins [CD63-Glycerol Kinase 5 (GLPK5)-Sphingomyelin Phosphodiesterase (SPHM)-Prostate-specific antigen (PSA)-Pappalysin-1 (PAPP)] could distinguish low-grade and high-grade PCa^[70]^. Bijnsdorp *et al*. identified two integrins (ITGA3) and Integrin subunit beta 1 (ITGB1) to be enriched in urine samples from metastatic PCa patients as compared to BPH or primary PCa^[71]^. MicroRNA contents of EVs have been found to be of diagnostic and prognostic potential in PCa. Huang *et al*. examined potential miRNA biomarkers for CRPC and identified a combination of EV-associated miR-1290 and miR-375 to be of prognostic significance, with their high levels correlating with poor clinical outcomes^[72]^. In another study, EV-associated miR-423-3p was found as a potential biomarker for prediction of castration-resistance^[73]^.

Authors contribution: To identify non-invasive biomarkers for therapy-induced NED in CRPC patients, authors examined EVs as a source of miRNA and protein-based markers^[74]^. Towards this, the authors performed small RNA next-generation sequencing in serum EVs isolated from a cohort of CRPC patients with adenocarcinoma characteristics (CRPC-Adeno) *vs.* those with NE features (CRPC-NE) was performed. This analysis identified a specific pattern of miRNA alterations in EVs that included a significant dysregulation of 182 known and four novel miRNAs in CRPC-NE. Further, machine learning algorithms could develop an “EV-miRNA classifier” that could robustly stratify “CRPC-NE” from “CRPC-Adeno”. Importantly, two miRNAs, miR-28-5p and miR-148a-3p, could distinguish between CRPC-Adeno and CRPC-NE states with high specificity and sensitivity. Examination of the protein repertoire of exosomes from CRPC-NE models by mass spectrometry identified several dysregulated proteins. Importantly, several heat shock proteins, such as HSP70, HSP90 were downregulated in NEPC, while thrombospondin 1 (TSP1) was upregulated^[74]^. Thrombospondin-1 is an anti-angiogenic factor that has been reported to be repressed in NEPC^[75]^. Furthermore, EV-associated BRN4 and BRN2 are potential novel non-invasive biomarkers to predict the emergence of NED in late-stage PCa^[51]^. These non-invasive EV markers can provide significant advance over existing methods of assessing NED based on histopathological criteria that are often flawed owing to the heterogeneity of NED^[10,11]^. An assessment of EV-based miRNA biomarkers for distinguishing between indolent and aggressive PCa identified miR-1246 as a potential non-invasive biomarker for aggressive PCa^[76]^ using Nanostring technology. Studies showed that miR-1246 is a tumor suppressive miRNA that is selectively released from PCa EVs, qualifying its potential utility as a biomarker for aggressive PCa.

Collectively, these studies position EV-based assays as a versatile liquid biopsy platform with expanding utility across early detection, disease risk stratification, and longitudinal treatment monitoring in PCa^[62]^.

### Exploiting EVs for the treatment of prostatic diseases

EVs, including exosomes and microvesicles, have emerged as promising tools for the treatment of prostatic diseases such as BPH and PCa. These nanoscale vesicles offer a natural delivery system that has low toxicity, low immunogenicity, and good permeability. Membrane association and endocytosis of exosomes enable both near-range and long-range intercellular signaling. There are four main categories of modifications that can be applied to exosomes to efficiently target and deliver to tumors, including biological, immunological, physical, and chemical modifications^[77]^. Modifications include altering peptides, antibodies, magnetic particles, and sodium bicarbonate, respectively^[77]^. Based on the target tissue and location, specific modifications can be made to enable efficient delivery of the engineered exosomes. Most often, multiple alterations and modifications are made to exosomes so that their ability to target tissue or cells is further enhanced^[77]^. Furthermore, exosomes can be targeted to specific cell types using antibodies against the surface antigens and can be modified to induce tumor cell killing. Studies have exploited these approaches and have used EVs for the treatment of benign conditions such as BPH^[78]^ to CRPC^[79]^ and aggressive, lethal PCa variants, NEPC^[13]^. For BPH, plant-derived nanovesicles from pomegranate juice (Punica Granatum) (referred to as “POM-NVs”) were used and were found to possess tremendous therapeutic potential to reverse BPH-associated changes *in vitro* and *in vivo*^[78]^. BPH has been associated with the induction of EMT^[80-82]^, mediated via Transforming Growth Factor beta (TGF-β)-Suppressor of Mothers Against Decapentaplegic (Smad signaling)^[82]^. An elevation in bone morphogenic protein 5 (BMP5) signaling has been reported to be a cardinal alteration driving BPH^[5]^. A member of the TGF-β family, BMP5 has been shown to be elevated, representing a cardinal molecular alteration in BPH^[83]^. Notably, POM-NVs could reverse TGF-β-mediated EMT and attenuate BMP5 signaling, leading to the restoration of prostate epithelial states *in vivo* and *in vitro*. Considering the large incidences of BPH and lack of effective therapies for clinical management, administration of POM-derived vesicles could provide a natural, economical method to improve the clinical management of BPH.

**Figure 5:**
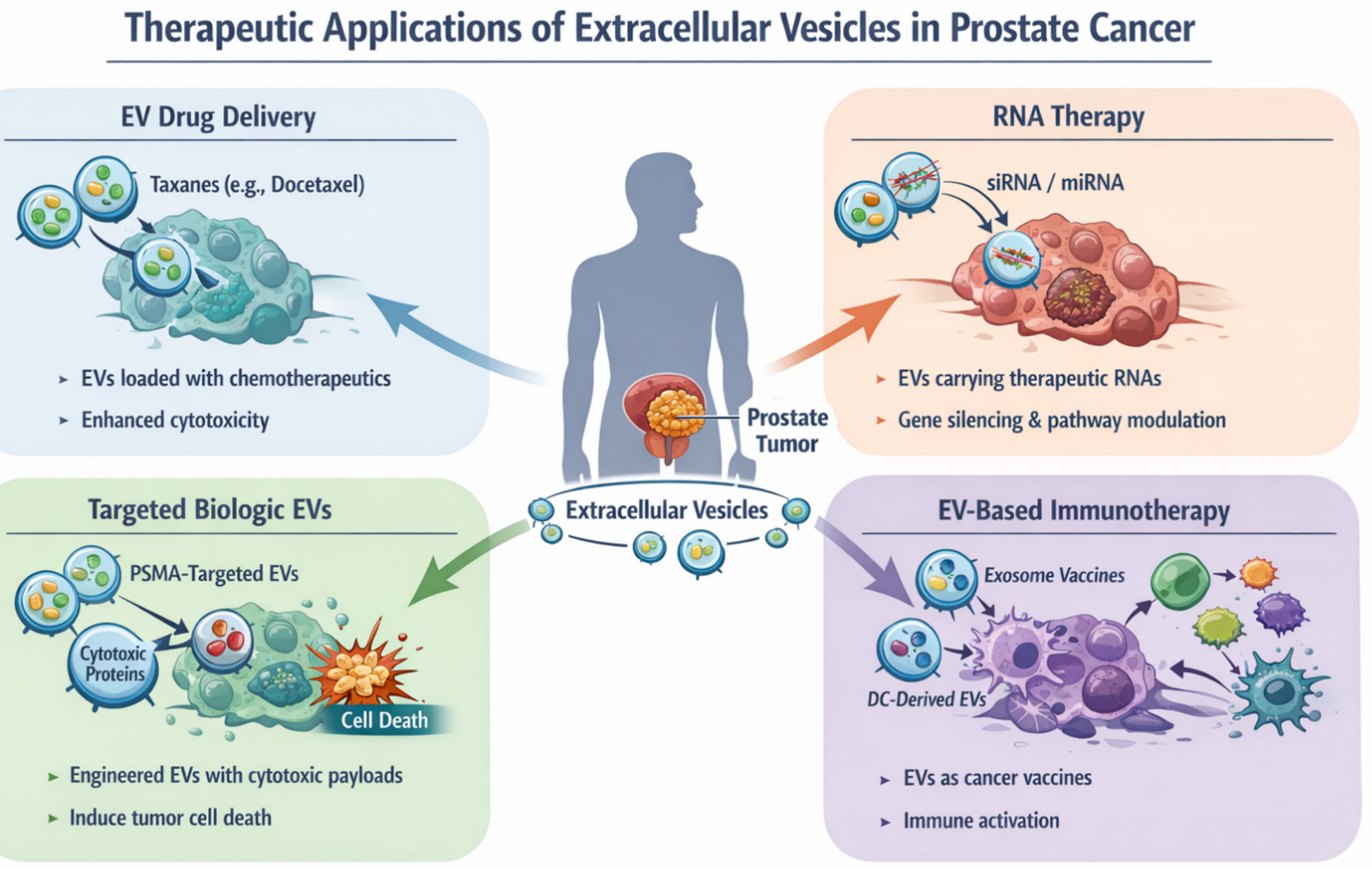
Therapeutic application of EVs in prostate cancer. EVs have been employed for delivery of cytotoxic drugs, targeted biologics, siRNA/miRNA or for immunomodulation in CRPC. This figure is a conceptual illustration created using an AI-assisted illustration tool for visualization purposes and does not represent experimental data. EVs: Extracellular vesicles; siRNA: small interfering RNA; miRNA: microRNA; PSMA: prostate-specific membrane antigen; DC: dendritic cell; CRPC: castration-resistant prostate cancer.

At the other end of the spectrum of prostatic diseases, EVs have been successfully engineered to therapeutically target CRPC and NEPC tumors^[13,79]^. EVs have been used as drug delivery vehicles for cytotoxic drugs, cytotoxic proteins/biological payloads or delivery of specific RNAs [Figure 5]. Saari *et al.* employed EVs isolated from LNCaP and PC3 cells to effectively deliver paclitaxel to autologous PCa cells^[84]^. Diao *et al.* used functional membrane-penetrating peptide Tyrosine Aminotransferase (TAT) as an effective natural means to deliver small interfering RNAs (siRNAs) against *FLOH1*, *NKX3*, and *DHRS7* genes into EVs^[79]^. The simultaneous siRNA-mediated knockdown of *FLOH1*, *NKX3*, and *DHRS7* genes via these engineered EVs led to increased apoptosis of LNCaP-AI (androgen independent) cells, suggesting a potential for improving CRPC treatment^[79]^. EVs have also been employed to induce immune responses in PCa. In a recent study, ScFv fragment of PSMA antibody was displayed on HEK293T exosomes and were loaded with gasdermin (GSDMD), a pyroptosis initiator to induce immune responses and inhibit tumor growth in PCa mouse models^[85]^. Interestingly, EV-based approaches have been examined as “cell-free vaccines” for PCa. Fan *et al*. employed EVs isolated from mature dendritic cells (DCs), loaded with cDC1-specific chemokine XCL1 and tested them in combination with chemotherapeutic agent cisplatin. This approach significantly increased the number and cytotoxic activity of CD8+ T cells, improved the tumor microenvironment leading to suppression of PCa growth^[86]^.

**Figure 6:**
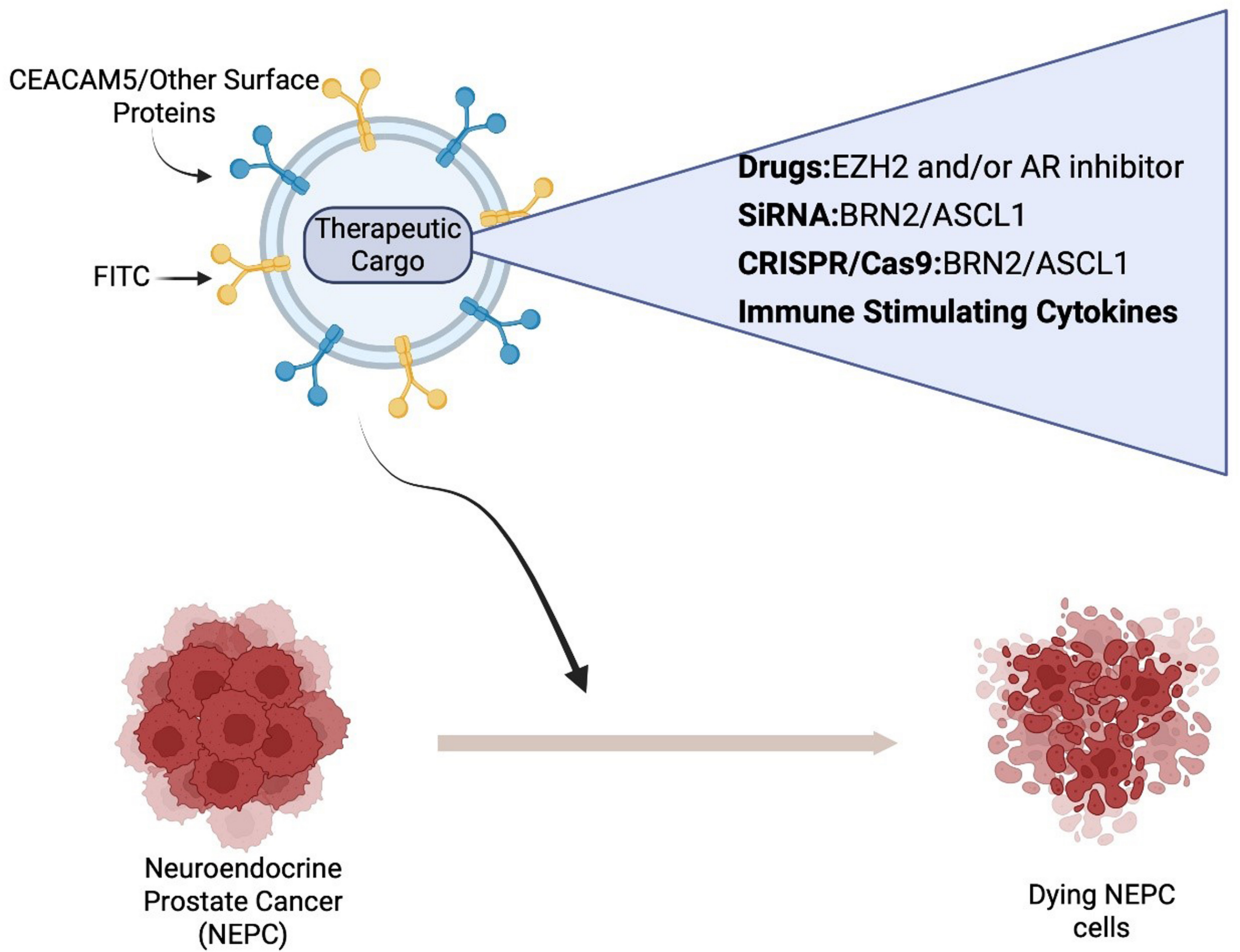
Schematic representation of EV mediated therapy against NEPC. EVs can be designed to target CEACAM5/other surface antigens and loaded with a variety of therapeutic cargo. Created in BioRender. Saini, S. (2026) https://BioRender.com/orbnahz. EVs: Extracellular vesicles; NEPC: neuroendocrine prostate cancer; siRNA: small interfering RNA; CRISPR/Cas9: clustered regularly interspaced short palindromic repeats/CRISPR-associated protein 9; CEACAM5: carcinoembryonic antigen-related cell adhesion molecule 5; EZH2: enhancer of zeste homolog 2; AR: androgen receptor; ASCL1: achaete-scute family bHLH transcription factor 1; BRN2: brain-specific homeobox/POU domain protein 2; FITC: fluorescein isothiocyanate.

Authors Contribution: For NEPC, exosomes were modified to display antibodies against the cell surface marker Carcinoembryonic antigen (CEACAM5)^[87]^, an antigen overexpressed on NEPC cells. To design CEACAM5 targeting exosomes (CTEs), a modular EV membrane anchoring platform consisting of streptavidin (STVDN) conjugated with 1,2-bis (dimethylphosphino) ethane: polyethylene glycol 5k (DMPE-PEG), referred to as DMPE-PEG-STVD, was employed to attach biotin-labelled CEACAM5 antibody^[87]^. To allow for tracking of exosomes, biotin-Fluorescein isothiocyanate (FITC) was incorporated on the exosome surface as well. CTEs were loaded with drugs Tazemetostat + ENZ, an EZH2 inhibitor^[34]^, and an AR inhibitor. Enhancer of zeste homolog 2 (EZH2), the catalytic subunit of Polycomb repressive complex 2 (PRC2), is overexpressed in NEPC^[7,16-18]^. While AR expression is largely inhibited in NEPC, residual AR activity has been shown to cooperate with EZH2 in driving neuronal gene programs in NEPC^[20]^. Data obtained using the NEPC patient-derived xenograft model LuCaP145.1 demonstrate that CEACAM5-engineered exosomes were targeted specifically to the NEPC cells. Loading of the exosomes with tazemetostat and ENZ resulted in NEPC tumor regression *in vivo*, supporting its feasibility as a novel treatment for NEPC^[13]^. These studies highlight the versatile application of EVs for the therapy of prostatic diseases. Targeting antibodies against surface antigens on exosomes can be expanded for other surface antigens in PCa [Figure 6]. Importantly, EVs are versatile and have high payload capacity and can be loaded with a variety of cargo, including drugs, siRNAs, CRISPR/Cas9 components, and proteins, allowing for combination therapies. For NEPC, therapeutic cargo can potentially include chemotherapeutic drugs or siRNAs/CRISPR-Cas9 components against transcription factors such as BRN2, and ASCL1 [Figure 6]. Alternatively, we propose the delivery of immune-stimulating cytokines to induce immune responses in immunologically cold NEPC tumors. The non-immunogenicity of EVs, easy scalability of EV isolation processes, and low cost of EVs support a potential easy translation to humans. Future studies are needed to examine these potential therapeutic strategies against this lethal variant of PCa. Methodological limitations in the study of EVs in PCa: Despite advances in understanding of EV-mediated communication in PCa, inherent challenges of the field remain. The tremendous heterogeneity of EV sub-populations is a central limitation as studies often rely on operationally defined preparations rather than subtypes that are validated based on their biogenesis. Further, various EV isolation methods such as differential ultracentrifugation, size exclusion chromatography, and affinity-based methods each enriches distinct subpopulations of EVs, limiting cross-study comparability. The lack of standardized methods for isolation, quantification of EVs and normalization contributes to variability and hampers reproducibility across laboratories. Purity concerns, such as, co-isolation of lipoproteins and protein complexes, are further confounders. To promote rigor, reproducibility, and standardization in EV-based biomarkers, these challenges need to be addressed. Further, scalable EV-based assays need to be developed using appropriate controls, references, and standards. There is a lack of spatial or *in situ* evidence directly demonstrating EV-cell interactions within intact tissues. Most of the studies rely on “*in vitro*” uptake assays or systemic delivery models. Addressing these methodological gaps is essential for improving rigor, reproducibility and biological interpretations in the field.

### Conclusions and future perspectives

In summary, EVs have emerged as critical mediators of intercellular communication in both benign and malignant prostatic diseases. In BPH, EVs contribute to stromal-epithelial crosstalk and inflammation, potentially influencing tissue remodeling and disease progression. In PCa, tumor-derived EVs facilitate key processes including immune evasion, androgen independence, metastasis, and resistance to therapy by delivering oncogenic proteins, RNAs, and signaling molecules to recipient cells. Despite growing recognition of EVs as key mediators of disease progression, their precise role remains incompletely defined. For NEPC, further research efforts are needed to understand the contributions of EV-mediated communication in this intricate process, which is key to elucidating the mechanistic basis of NE state emergence. Such an understanding is needed to better understand the drivers of the disease, with implications for the development of better targeted therapies. Current studies in this area are limited by the scarcity of bona fide NEPC models and patient cohorts, making it difficult to distinguish EV-specific signatures that truly reflect NE transformation. Further, circulating exosomes represent a heterogeneous mixture of vesicles released from tumor, stromal, and immune cells. Therefore, attributing functional effects to tumor-derived vesicles remains a major technical challenge. Standardized methods for isolation of tumor-derived EVs followed by characterization are needed to circumvent these issues. Considering the methodological limitations in EV research, we are still far away from a comprehensive understanding of the role of EVs in CRPC and in driving lineage switching. Such an understanding would require systematic, concerted investigations using standardized EV isolation methods, characterization, and *in situ* techniques.

Clinically, EVs represent a promising class of non-invasive biomarkers that aid in early detection, risk stratification, and treatment monitoring. Despite promising initial findings of EV-based markers, there remain challenges in standardizing EV isolation methods as well as validating biomarkers for clinical use. Multi-omics studies with EVs are likely to be more useful in cancer diagnosis and prognosis^[88]^. Therapeutically, engineered EVs offer an innovative platform for the targeted delivery of cytotoxic drugs, small molecules, and immune modulatory agents, particularly for CRPC and treatment-resistant subtypes such as NEPC. Though therapeutic EV studies remain predominantly pre-clinical, but several proof-of-concept studies demonstrate a promising potential of EV-based therapeutics for late stage PCa.

Looking ahead, integrating EV profiling with spatial transcriptomics and single-cell analytics may further refine our understanding of disease heterogeneity and progression. Rigorous research is thus necessary to translate these scientific breakthroughs into routine clinical practice. Future research will also focus on optimizing EV production, cargo loading, and targeting strategies to unlock their full potential as next-generation diagnostic and therapeutic tools in prostate disease management. Addressing these challenges and continued collaboration between basic scientists and clinicians are needed. Further, early contact with regulatory agencies during EV-based assay development can remove regulatory bottlenecks. In conclusion, we have made significant progress in the EV field in PCa. However, several roadblocks remain that prevent clinical translation of promising EV-based biomarkers and therapeutics in PCa and other prostatic conditions.

## DECLARATIONS

### AI and AI-assisted tools statement

AI was used for literature review of some parts. However, AI was not used for writing the article. It was only used as a tool for literature review. In addition, Figure 5 and Table 1 was generated by AI tool (Chatgpt). All AI-generated content was critically reviewed, edited, and approved by the authors, who assume full responsibility for the final figures.

### Copyright

© The Author(s) 2026.
